# Superstitious Remedies

**Published:** 1893-04

**Authors:** 


### SUPERSTITIOUS REMEDIES.

Among the ancients and among savages we expect to find supestitious remedies in vogue. In Pliny's day it does not seem unnatural that the accepted cure for certain maladies, believed in by the educated and ignorant alike, should be a paste made of crushed snails found in the ruts of the road and gathered at a certain hour in the morning; nor that other ills were supposed to be cured by touching an elephant, the cure being the more swift and sure if at the moment of being touched the great beast should chance to sneeze.

But it is quite another thing when the children of our own public schools believe that to touch a toad will cause warts, and that when such warts appear the proper way to cure them is to tie a bit of toadstool with red thread upon the afflicted hand for three nights in succession.

Another wart cure commonly practised among children is still more fantastic and absurd. It consists in pricking the wart until it bleeds, allowing the blood to drop upon a penny and then throwing the penny away; whoever picks up the penny will "get the wart"—that is, a wart will appear on the hand of that person, and at the same time the original wart will disappear from the hand of the first sufferer.

Such a belief as this seems to belong naturally in the Middle Ages. Yet the children who in the nineteenth century and the United States try to rid themselves of warts by a charm for transference can be numbered by thousands.

But besides the superstitions long current among ignorant Americans regarding cures, new ones are often imported by immigrants from other lands. A little school girl who had the jaundice, and whose skin was consequently very yellow, was recently advised by a Norwegian acquaintance what to do.

"It is of no use to have a doctor," she was told. "A doctor cannot do anything for the jaundice; but my mother has told me how you can be cured. You must boil a yellowbird and eat his meat and the soup made from his flesh; that will cure you. If you cannot get a yellowbird, then take yellow silk—it is the yellow you must have—and boil that and drink the yellow water, and you will get well."

This advice was not taken, but nevertheless the little girl speedily recovered under the care of a skillful physician.—*Youth's Companion.*

